# Nicotinamide-N-Methyltransferase gene rs694539 variant and migraine risk

**DOI:** 10.1186/s10194-016-0688-8

**Published:** 2016-10-10

**Authors:** Ali Sazci, Gensay Sazci, Bilgen Sazci, Emel Ergul, Halil Atilla Idrisoglu

**Affiliations:** 1 Department of Medical Biology and Genetics, Faculty of Medicine, University of Kocaeli, Kocaeli, 41380 Turkey; 2 Department of Neurology, Istanbul Faculty of Medicine, University of Istanbul, Istanbul, Capa, 34290 Turkey

**Keywords:** NNMT gene, Association, rs694539, One carbon metabolism, Gender association, Turkey

## Abstract

**Background:**

Migraine is a common neurovascular disorder affecting 10 to 20 % of the world population usually subdivided into migraine with auro (MA) and migraine without auro (MO). Homocysteine is involved in the pathophysiology of a number of neurological disorders. Elevated levels of homocysteine in the plasma is produced by the MTHFR gene rs 1801133 and rs 1801131 variants as well as the NNMT gene rs 694539 variant.

**Methods:**

With the polymerase chain reaction-restriction fragment length polymorphism method developed recently in our laboratory, we were able to show an association between the NNMT gene rs694539 variant and migraine for the first time.

**Results:**

Here we report the association of the Nicotinamide-N-methyltransferase gene (NNMT) rs694539 variant with migraine in a case–control study of 433 patients with migraine and 229 healthy controls (*χ*2 = 6.076, *P* = 0.048). After stratification, we were able only to show an association between the NNMT gene rs694539 variant and female patients with migraine on the genotype and allelic levels. However there was no association in male patients with migraine (*χ*2 = 1.054, *P* = 0.590).

**Conclusions:**

Consequently our results clearly indicate that the NNMT gene rs694539 variant is a genetic risk factor for migraine.

## Background

Migraine is a common and chronic neurovascular disorder affecting approximately 10–20 % of the world population. Clinically, It is sub-classified into migraine with aura (MA) and migraine without aura (MO) accompanied by severe recurrent headache attacks and associated symptoms such as nausea, vomiting, photo- and phonophobia [1].

Homocysteine, a sulfur-containing amino acid derived from the metabolism of methionine, has been implicated in the pathophysiology of a variety of neurological disorders such as migraine [1], essential tremor [2], stroke [3], epilepsy [4]), schizophrenia [5, 6] and bipolar disorders [7, 8]. 5,10-Methylenetetrahydrofolate reductase (MTHFR), a key enzyme in the metabolism of folate catalyzes the reduction of 5,10-methylenetetrahydrofolate to 5-methyltetrahydrofolate, the predominant form of folate and carbon donor for the remethylation of homocysteine to metionine. Variants of the MTHFR C677T (rs1801133) and A1298C (rs1801131) gene, associated with significantly elevated plasma homocysteine levels, were shown to be associated with migraine [9–21]. A meta-analysis has revealed that the MTHFR C677T (rs1801133) variant is a genetic risk factor for migraine [22]. Another enzyme implicated in one-carbon metabolism is nicotinamide-N-methyltransferase (NNMT). Human NNMT (EC 2.1.1.1), a cytoplasmic enzyme belonging to Phase II conjugating enzymes, is reported to be expressed in brain and other nervous tissues [23]. This gene is 16.703 bp in lenght on chromosome 11q23.1, having three exons and two introns. NNMT catalyzes the transfer of methyl group from S-adenosyl-L-methionine (SAM) to nicotinamide (NA), thus creating 1-methylnicotinamide (1MNA) and S-adenosylhomocysteine (SAH) which is later hydrolyzed to homocysteine [24]. Homocysteine is one of the key components of one-carbon metabolism. The rs694539 NNMT variant, localized at the 114133419 bp (G > A transition) is found to be significanly associated with elevated plasma homocysteine levels [25]. Elevated plasma homocysteine levels were shown to be associated with an increased risk of migraine in patients with the MTHFR C677T and A1298C polymorphisms [1, 11].

Recently, we showed that the MTHFR C677T and A1298C variants were associated with migraine [1]. We also wanted to reveal whether the rs694539 variant of NNMT gene is associated with migraine. To do so we analyzed the allele and genotype frequencies of the NNMT gene rs694539 variant in 433 patients with migraine and 229 healthy controls.

## Methods

### Subjects

The subjects were 433 [351 (81 %) female; 82 (19 %) male] patients with migraine and 229 [184 (80 %) female; 45 (20 %) male] healthy controls recruited from the Istanbul University neurological clinic. The mean age of migraine was 36.96 ± 14.863 years and controls was 37.54 ± 16.403 years; age range for cases was 18–89 years, and for controls was 19–85 years. All patients were diagnosed by an experienced neurologist using International Classification of Headache Disorders (ICHD) the third version (2013) [26]. The institutional review board approved the present study and informed consent was obtained from all subjects.

### Genotyping

Genomic DNA was isolated from whole blood using the salting-out procedure [27]. Genotypes of the subjects were determined using a polymerase chain reaction-restriction fragment length polymorphism (PCR-RFLP) method developed in our laboratory [28].

### Statistical analysis

The Hardy-Weinberg equilibrium was verified for both cases and controls. Differences between cases and controls were determined using the *χ*2 test and Student’s *t* test. Genotype and allele frequencies were calculated using the *χ*2 test. Odds ratio (OR) and confidence interval (95 % CI) were estimated using 2x2 cross-tabulation and a binary logistic regression model for age and gender. All statistical analyses were done using SPSS software package version 21.0. The *P* value <0.05 % was taken as significant.

## Results and discussion

Here, we report an association between the rs694539 variant of NNMT gene with migraine for the first time (*χ*2 = 6.076, *P* = 0.048). The statistical power for all cases is 0.40 and for overall controls is 0.24 (Table 1). The individuals with AA genotype confer a 3.8-fold increased risk for migraine (*χ*2 = 5.372, *P* = 0.020, OR = 3.840, 95 % CI = 1.133–13.013). Moreover, the G allele showed a 3.8-fold protection against migraine (*χ*2 = 5.372, *P* = 0.020, OR = 0.260, 95 % CI = 0.077–0.883). Stratification analysis according to gender revealed that there was an association only in female patients with migraine. The female individuals with the AA genotype revealed a 4.3-fold increased risk for migraine (*χ*2 = 4.474, *P* = 0.034, OR = 4.346, 95 % CI = 0.988–19.112). Similarly the individuals with the G allele showed a 4.3-fold protection against migraine (χ = 4.474, *P* = 0.034, OR = 0.230, 95 % CI = 0.052–1.012). However there was no association in male patients with migraine (*χ*2 = 1.054, *P* = 0.590). All patients with migraine and controls were in Hardy-Weinberg equilibrium (0.665 and 0.152, respectively) (Table 1).

**Table 1:** Genotype and Allele frequencies of the NNMT gene rs694539 variant in patients with Migraine and controls

Gene	Cases	Controls	*χ*2	*P*-Values	OR; 95 % CI
NNMT Rs694539	433 (100.0)	229 (100.0)	6.076	0.048	
GG	271 (62.6)	156 (68.1)	2.005	0.157	0.783 (0.558–1.099)
GA	141 (32.6)	70 (30.6)	0.275	0.600	1.097 (0.776–1.550)
AA	21 (4.8)	3 (1.3)	5.372	0.020	3.840 (1.133–13.013)
Allele frequency					
G Allele	683 (78.87)	382 (83.40)	5.372	0.020	0.260 (0.077–0.883)
A Allele	183 (21.13)	76 (16.59)	2.005	0.157	1.277 (0.910–1.794)
HWE(exact)	0.665	0.152			
Statistical power	0.40	0.24			
Gene	Cases Female	Controls Female	*χ*2	*P*-Values	OR; 95 % CI
NNMT rs694539	351 (100.0)	184 (100.0)	5.650	0.059	
GG	220 (62.7)	128 (69.6)	2.518	0.113	0.735 (0.502–1.076)
GA	115 (32.8)	54 (29.3)	0.652	0.419	1.173 (0.796–1.729)
AA	16 (4.6)	2 (1.1)	4.474	0.034	4.346 (0.988–19.112)
Allele frequency					
G Allele	555 (79.06)	310 (84.24)	4.474	0.034	0.230 (0.052–1.012)
A Allele	147 (20.94)	58 (15.76)	2518	0.113	1.361 (0.930–1.993)
HWE(exact)	0.872	0.262			
Statistical power	0.43	0.25			
Gene	Cases Male	Controls Male	*χ*2	*P*-Values	OR; 95 % CI
NNMT rs694539	82 (100.0)	45 (100.0)	1.054	0.590	
GG	51 (62.2)	28 (62.2)	0.000	0.998	0.999 (0.472–2.114)
GA	26 (31.7)	16 (35.6)	0.194	0.659	0.842 (0.391–1.813)
AA	5 (6.1)	1 (2.2)	0.969	0.325	2.857 (0.323–25.244)
Allele frequency					
G Allele	128 (78.05)	72 (80.00)	0.969	0.325	0.350 (0.040–3.092)
A allele	36 (21.95)	18 (20.00)	0.000	0.998	1.001 (0.473–2.119)
HWE(exact)	0.520	0.665			
Statistical power	0.05	0.04			

*HWE* Hardy-Weinberg Equilibrium

Distributions of genotypes GG, GA and AA were 62.6, 32.6 and 4.8 % in overall patients with migraine and 68.1, 30.6 and 1.3 % in overall controls respectively (Table 1). The frequencies of G allele were 78.87 % in overall cases and 83.40 % in overall controls (Table 1). The frequencies of A allele were 21.13 % in overall migraine patients and 16.59 % in overall controls (Table 1).

In the case control studies, a number of polymorphisms have been shown to be associated with migraine. Plasma urotensin-2 level and thr21met variant have been reported to be associated with migraine [29]. It has been also shown that the glutamatergic system is involved in the pathophysiology of migraine [30]. DNA methylation status of RAMP1 gene has been shown to be associated with migraine [31]. Rs4379368 in the C7orf10 gene and rs13208321 in the FHL5 gene have been reported to be associated with migraine [32]. Genetic variants in the SYNE1 and TNF genes have been shown to be related to menstrual migraine [33].

Recently genome-wide association studies have implicated neuronal, vascular, metalloproteinase and pain pathways in migraine [34].

More recently, GWASs have identified several single nucleotide polymorphisms associated with migraine pathophysiology [35–37] in genes and in regulatory regions of genes implicated in epigenetic processes, including MTDH, MEF2D and PRDM16. Metadherin (MTDH) is shown to be associated with nuclear factor B (NFB) and a HAT to promote the expression of NFB target genes [38]. Myocyte enhancer factor 2D (MEF2D) can target methyltransferase complexes to specific genes to make them available for gene expression [39]. MEF2 has recently been shown to be modulated through the glucocorticoid receptor [40], which may be one of the mechanisms by which stress hormones affect the epigenome. Finally, PR domain containing 16 (PRDM16) is implicated in positioning and removing specific chromatin modifications at enhancer regions of Notch target genes during olfactory neuron differentiation [41]. These studies indicate that some of the migraine GWAS hits may contribute to developing migraine through epigenetic alterations at their target genes [42].

Two GWAS have been carried out in migraine. One of which concentrated mainly on patients with migraine with auro and identified a single SNP in the MTDH gene [34]. The other study concentrated on migraine without auro and identified six SNPs in the MEF2D, TGFBR2, PHACTR1, ASTN2, TRPM8, and LRP1 gene associated with migraine [37]. Recently a meta-analysis of studies on migraine identified 12 SNPs significantly associated with migraine [42]. Thus far 13 migraine susceptibility loci identified are associated with neuronal (MTDH, LRP1, PRDM16, MEF2D, ASTN2, PHACTR1, FHL5, MMP16), vascular (PHACTR1, TGFBR2, c7orf10), metalloproteinase (MMP16, TSPAN2, AJAP1) and pain (TRPM8) pathways. Most recently, GWAS using 59 674 migraine cases and 316 078 controls identified 45 independent migraine associated SNPs mapping to 38 distinct genomic regions, 28 of which have not been previously reported [43].

However, global DNA methylation has not been reported in patients with migraine, elevated homocysteine levels were revealed to be associated with cognitive impairment [44, 45].

## Conclusions

In summary, the role of the rs694539 variant of the NNMT gene in migraine is unclear. Nonetheless, through dysregulation of epigenetics and/or elevated homocysteine levels or dysregulation of nicotinamide levels may result in migraine or particularly through vascular processes. Thus, our findings suggest that the rs694539 variant of NNMT gene may play a role in the etiopathology of migraine in the Turkish population studied herein.
